# HOFE: an interactive forensic entomological database

**DOI:** 10.1093/database/baae058

**Published:** 2024-07-13

**Authors:** Mandie Liu, Yihong Qu, Yifei Luo, Binta J J Jallow, Yuting Ma, Afito Luciano, Jingjing Huang, Jifeng Cai, Fanming Meng

**Affiliations:** School of Basic Medical Sciences, Central South University, Changsha, Hunan 410013, China; School of Basic Medical Sciences, Central South University, Changsha, Hunan 410013, China; School of Basic Medical Sciences, Central South University, Changsha, Hunan 410013, China; School of Basic Medical Sciences, Central South University, Changsha, Hunan 410013, China; School of Basic Medical Sciences, Central South University, Changsha, Hunan 410013, China; School of Basic Medical Sciences, Central South University, Changsha, Hunan 410013, China; Department of Forensic Medicine, Key Laboratory of Forensic Medicine, School of Basic Medical Sciences, Xinjiang Medical University, Urumqi, Xinjiang 830017, China; Xinjiang Key Laboratory of Molecular Biology for Endemic Diseases, Urumqi, Xinjiang 830017, China; School of Basic Medical Sciences, Central South University, Changsha, Hunan 410013, China; Department of Forensic Medicine, Key Laboratory of Forensic Medicine, School of Basic Medical Sciences, Xinjiang Medical University, Urumqi, Xinjiang 830017, China; Xinjiang Key Laboratory of Molecular Biology for Endemic Diseases, Urumqi, Xinjiang 830017, China; School of Basic Medical Sciences, Central South University, Changsha, Hunan 410013, China; Department of Forensic Medicine, Key Laboratory of Forensic Medicine, School of Basic Medical Sciences, Xinjiang Medical University, Urumqi, Xinjiang 830017, China; Xinjiang Key Laboratory of Molecular Biology for Endemic Diseases, Urumqi, Xinjiang 830017, China

## Abstract

The significance of entomological evidence in inferring the time, location and cause of death has been demonstrated both theoretically and practically. With the advancement of sequencing technologies, reports have emerged on necrophagous insects’ nuclear genomes, transcriptomes, proteomes and mitochondrial genomes. However, within the field of forensic entomology, there is currently no available database that can integrate, store and share the resources of necrophagous insects. The absence of a database poses an inconvenience to the application of entomological evidence in judicial practice and hampers the development of the forensic entomology discipline. Given this, we have developed the Home Of Forensic Entomology database, encompassing 10 core functional modules: Home, Browse, Mitochondria, Proteome, JBrowse, Search, BLAST, Tools, Case base and Maps. Notably, the ‘Tools’ module enables multiple sequence alignment analysis (Muscle), homologous protein prediction (Genewise), primer design (Primer), large-scale genomic analysis (Lastz), Gene Ontology and Kyoto Encyclopedia of Genes and Genomes enrichment analysis, as well as expression profiling (PCA Analysis, Hcluster and Correlation Heatmap). In addition, the present database also works as an interactive platform for researchers by sharing forensic entomological case reports and uploading data and material. This database provides potential visitors with a comprehensive function for multi-omics data analysis, offers substantial references to researchers and criminal scene investigators and facilitates the utilization of entomological evidence in court.

**Database URL**: http://ihofe.com/

## Introduction

The main goal of forensic entomology is to determine the time, cause, manner and place of death under investigation, especially for severely decomposed corpses or skeletonized human remains, based on elements that can be inferred from insect studies found on or near corpses (1–3). As the field of application continues to expand, entomological evidence can also play an essential role in non-death investigations, such as drug abuse, linking suspects to crime scenes, child neglect, sexual harassment and identification of suspects (4–7).

Typically, the primary way to estimate the postmortem interval (PMI) of a decaying corpse involves determining the developmental age of necrophagous insects in their non-adult stages (i.e. eggs, larvae, or pupae) (8). For bodies that exceed a single generation of necrophagous insects, the primary method for estimating the PMI is the assessment of succession patterns, including the sequence of arrival and colonization times of different necrophagous insects on the corpse (9). Commonly, necrophagous flies of Calliphoridae, Sarcophagidae, Phoridae, Muscidae (Diptera), etc., are the first insect groups to discover and colonize the body, followed by beetles of Silphidae, Histeridae, Dermestidae, etc. (10–12). Accurate species identification represents the initial step in achieving a precise estimation of the minimum PMI (minPMI), and it is equally important to have a comprehensive understanding of the life cycles of these species (13–15). Morphological identification is widely utilized in forensic investigation. In some certain situations, molecular biology methods can achieve more precise identification (16). In 1994, Sperling *et al*. used DNA-based methods for species identification of insects with forensic importance for the first time (17). Nowadays, mitochondrial and nuclear DNA markers of these insects have been extensively studied (18–20). Of the mitochondrial genome, the *COI* (cytochrome c oxidase subunit I) gene is most commonly used in the molecular identification of necrophagous insects (21–23). The other markers like *COII*, ribosomal 16S rRNA, ribosomal 12S rRNA, *ND5* (reduced nicotinamide adenine dinucleotide dehydrogenase subunit 5), *Cytb* (cytochrome b), etc., are also used for species identification at the different taxonomical level by a single or combined way (24, 25).

Several *de novo*-assembled blowfly genomes have been reported in recent years, providing a deeper understanding of physiological and behavioral features. Using the Illumina sequencing platform, the draft genome of *Lucilia cuprina, Musca domestica* and *Phormia regina* were assembled (26–28). In 2020, the first chromosome-scale genome assembly of a necrophagous fly, *Aldrichina grahami*, was completed by the use of the short-read Illumina and long-read Pacific Biosciences (PacBio) sequencing platforms, which provides a robust genome reference for forensically important fly species (29). In 2021, the genome of *Sarcophaga peregrina* was sequenced and assembled using the PacBio platform (30). These genome resources serve as valuable references for transcriptomic analysis, aiding in the identification of candidate genes associated with necrophagous behavior in forensic insects (31, 32). For example, olfactory transcriptome analysis of *A. grahami* not only helps to explore the relationship between specific genes and volatile organic compounds (VOCs) but also facilitates the analysis of the olfactory differences and colonization preferences among different necrophagous species (33). The gene expression profile obtained from the developmental transcriptome of *S. peregrina* at each age stage provides a more precise method for estimating species’ age, achieving accurate inference of PMI (34). Moreover, their genetic family feature can be analyzed by combining genomic information from multiple species, providing insights into differences in foraging behavior and living habits of necrophagous insects living in different habitats (35). Many characteristics of necrophagous species, such as stress resistance and detoxification abilities, still need to be explored and studied at the genetic level (28, 36). Further integration of these scattered genetic resources is beneficial for in-depth research on necrophagous insect feeding behavior, lifestyle habits and developmental patterns.

Over the past decades, DNA databases have remained a focal point in the development of the forensic field. These forensic science DNA databases have played a key role in aiding millions of investigations conducted by judicial and law enforcement agencies (1). Some integrate extensive forensic clinical and demographic data, such as HOMED (homicides in eastern Denmark), a database containing information on all homicide cases investigated at the Department of Forensic Medicine in Copenhagen since 1971 (37). Given the nearly 100% medico-legal homicide autopsy rate within the department’s jurisdiction, the coverage of the database is deemed exceptionally comprehensive. Some offer identifiers for sequenced forensic alleles (short tandem repeats or single nucleotide polymorphism) and their microvariants for use in forensic allele nomenclature, such as FLAD (http://forensic.ugent.be/FLAD/) (38). Nowadays, many non-human genome databases, such as eggplant, orange, molluscan and panda (39–42), provide data for in-depth exploration of evolution and intraspecies variation (43). However, there is a lack of forensic non-human genetic data, both in forensic DNA databases and in commercial sequencing data, even in some species of high forensic importance like blowflies (44). Here, we unveil the Home Of Forensic Entomology (HOFE), an interactive database dedicated to gathering, storing, analyzing and visualizing genomic datasets for insects of forensic importance. The establishment and application of the database not only fill the gap of the database of necrophagous insects but also provide solid technical support with vital data resources and an open interactive platform for applying forensic entomology in judicial practice and scientific research.

## Materials and methods

### Database implementation

**Figure 1.**
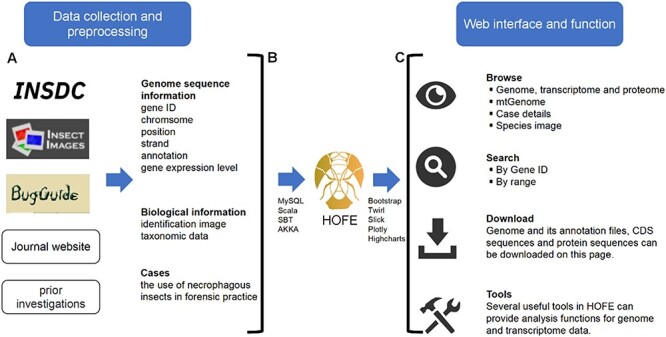
Schematic diagram of data processing for the HOFE. (**A**) Data collection and preprocessing. (**B**) Establishing data connections and implementing data indexing and storage using the MySQL database. (**C**) Outline of the web interface and function of HOFE.

The Linux operating system was utilized to deploy the interactive forensic entomology database, which was constructed employing Akka v2.12 (web server) (https://www.akka-technologies.com/) and MySQL v5.7.26 (database server) (https://www.mysql.com/), as shown in Figure 1. The MySQL database management system was employed for the processing and organization of all data. The design and execution were facilitated through the application of website interface components, including the Play Framework v2.6.25 (https://www.playframework.com/) and Bootstrap v3.3.0 (https://getbootstrap.com/). The query function was implemented using the Slick middleware layer v3.3.2. For visualization, box plots, bar plots, scatter plots and heatmaps were generated using Highcharts v9.0.1 (https://www.highcharts.com/). Shanghai BIOZERON Co., Ltd supported us in constructing the platform.

### Data source

The interactive forensic entomology database serves as a public platform for annotating, publishing and updating the genetic information data of necrophagous insects. The interactive forensic entomology database currently contains a genome-wide assembly of four highly forensically important species: *A. grahami*, *L. cuprina*, *M. domestica* and *S. peregrina*, transcriptomic and proteomic data information of *A. grahami*, as well as mitochondrion genomes of 13 necrophagous species. Among them, the genome assembly sequences of *L. cuprina*, *M. domestica* and *S. peregrina* and mitochondrial sequences of 13 species were manually extracted from the GenBank genome database of the National Center for Biotechnology Information (http://www.ncbi.nlm.nih.gov), European Bioinformatics Institute (https://www.ebi.ac.uk) and DNA Data Bank of Japan (https://www.ddbj.nig.ac.jp). While the genetic, transcriptomic and proteomic data resources of *A. grahami* were obtained from our previous work (29, 31), 12 823 protein-coding genes from the genome of *A. grahami* were annotated, with 5247 Kyoto Encyclopedia of Genes and Genomes (KEGG) pathways and 7518 Gene Ontology (GO) terms. The pupal stage, developmental and olfactory transcriptome and olfactory proteome of *A. grahami* were analyzed.

## Results

### Overview of HOFE

The database is named ‘Home Of Forensic Entomology’ (http://ihofe.com/) and primarily encompasses necrophagous taxa, including Diptera (Calliphoridae, Muscidae, Syrphidae, Sarcophagidae, Tachinidae, and Tabanidae), Coleoptera (Dermestidae, Silphidae, Histeridae, and Staphylinidae) and Hymenoptera, among others.

Registered users of the database are granted access to online queries for mitochondrial and nuclear genomic information and annotation details related to various necrophagous insects. This platform facilitates genome data visualization, sequence alignment submissions and download of pertinent data files. Additionally, it offers access to classification information and images for required species, enabling species identification. The platform also integrates a diverse array of bioinformatics tools, intended for retrieving gene function and location, analyzing structural features, collinearity and phylogenetics of necrophagous insect genomes. The case repository within the platform serves as a template for the forensic application of necrophagous insects.

### Database homepage

**Figure 2.**
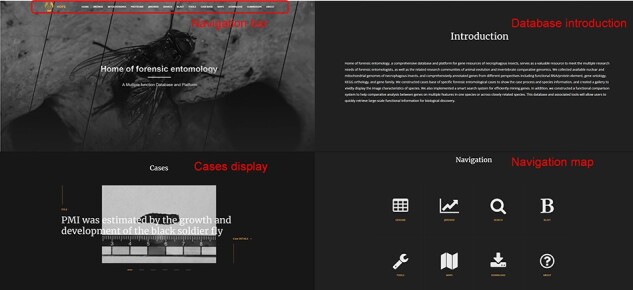
HOFE home page.

The interactive forensic entomology database is presented as a web portal. The database contains an intuitive navigation section that shows the main features, such as Browse, JBrowse, BLAST, Tools, Case base, etc. Below the navigation is information about the site, including a brief introduction, case presentations and navigation maps (Figure 2).

### Browse

**Figure 3.**
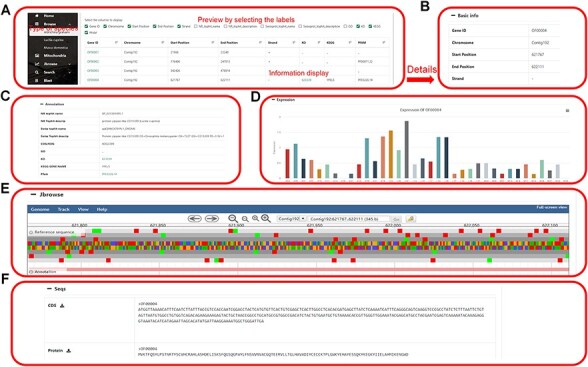
Data browsing of the HOFE. (**A**) Selected species with the following detailed genome information. (**B**) Basic information of the genome. (**C**) Annotation information of the genome. (**D**) Expression of the gene. (**E**) Visual genome browser and coding genes of the genome. (**F**) Download of CDS sequence and protein sequence.

The genome information section displays the genome information in the database, including the name of the species genome. Clicking on any gene will jump to the specific information webpage of the gene. This page includes basic information (gene number, chromosome, start site, stop site, plus strand and minus strand), genome annotation (COG/KOG, GO, InterProscan, KO, KEGG Symbol, and KEGG) of the gene, visualization of gene expression, JBrowse links and gene sequences download (Figure 3).

### JBrowse

**Figure 4.**
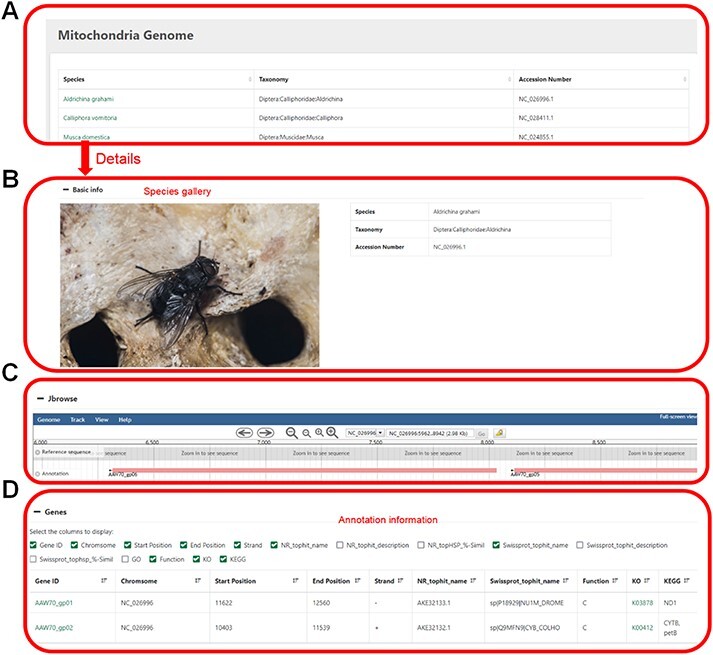
Mitochondrial genome information. (**A**) Selected species by name, taxonomic category or accession number. (**B**) Basic information of the mitochondrial genome. (**C**) Visual genome browser and coding genes of the mitochondrial genome. (**D**) Annotation information of the genome.

The database supports JBrowse; users can select the data track of interest in the selection box, in the right display area can zoom in and drag the genome, click on any gene to get the detailed information of the gene. You can also download the required sequence in the lower left drop-down bar, convenient for retrieval of gene sequence and location information (Figures 3E and 4C). Comparisons between species can also be viewed to provide a reference for genomic studies.

### Mitochondria

Users can click on the species name to enter the details page, which displays the sample image of the species, the mitochondrial genome information and all the coding genes with their annotations (Figure 4).

### Proteome

**Figure 5.**
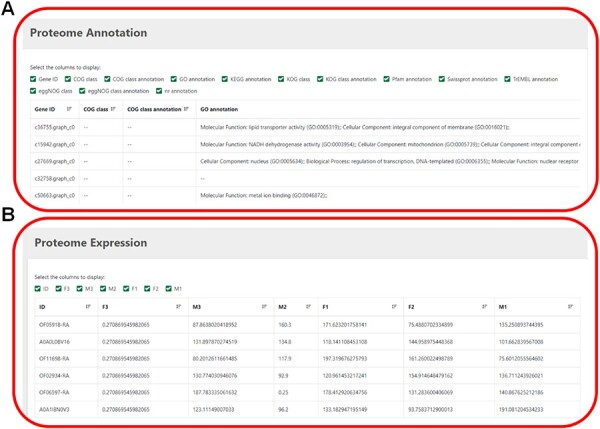
(**A**) The annotation information of proteome. (**B**) The expression level of proteome.

This module displays proteome annotation information and expression-level information (Figure 5). Several databases, including COG, CO, KEGG, KOG, Pfam, SwissProt eggNOG and nr, provide more detailed and comprehensive annotation of protein functions. Users can tick the desired gene ID to know its expression level.

### Search

**Figure 6.**
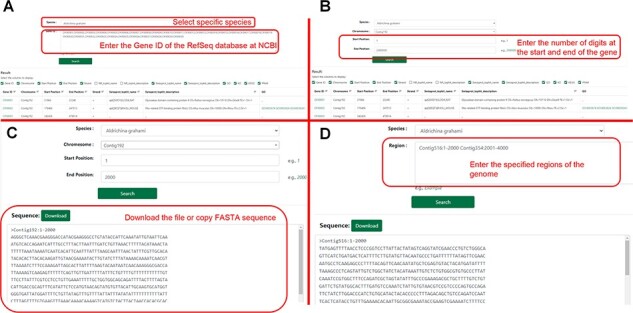
Detailed description of the ‘Search’ module. (**A**) Example of ‘Gene Search’ by ID. (**B**) Example of ‘Gene Search’ by range. (**C**) Example of ‘Sequence Fetch’. (**D**) Example of ‘Multiple Sequence Fetch’.

The search module contains two sub-pages, and users can search by gene ID or range to obtain gene information. The user can click ‘Sequence Fetch’ to download the nucleotide sequence of the specified region. They could enter the start and end points of the sequences in the search box and click ‘Download’ to gain all sequences within that range. This method can simultaneously obtain one or more gene sequences (Figure 6).

### BLAST

**Figure 7.**
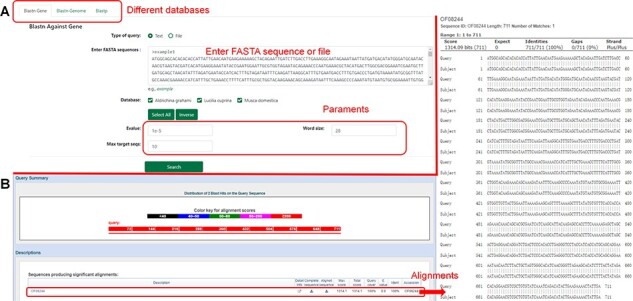
Introduction to the ‘Blast’ function. (**A**) Nucleotide sequences in the database compared using ‘Blastn’. (**B**) Results of nucleotide sequence alignment.

The database provides a sequence alignment tool (BLAST), which allows users to input or upload nucleic acid sequences or protein sequences in file form and select omics data in the database for comparison (Figure 7).

### Tools

**Figure 8.**
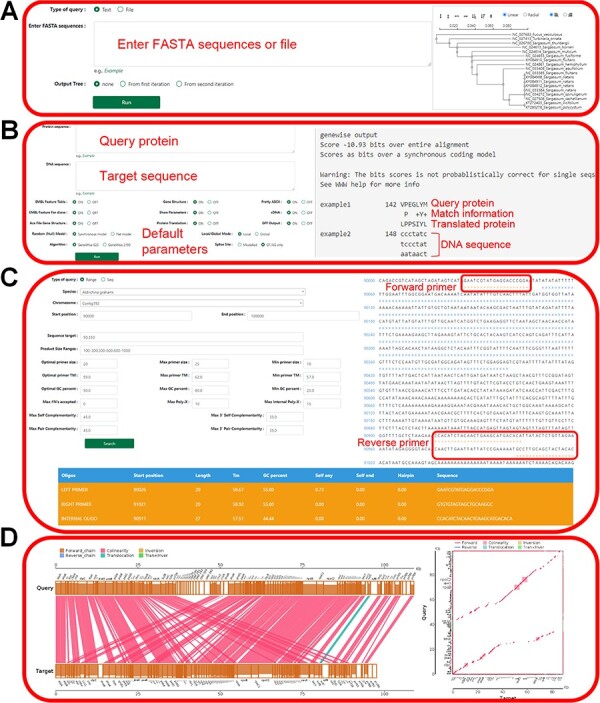
‘Tools’ of the HOFE. (**A**) ‘Muscle’ displaying alignment results and the phylogenetic tree. (**B**) ‘Genewise’ showing the prediction of homologous proteins. (**C**) Primer design. (**D**) ‘Lastz’ displaying the results of gene collinearity analysis.

**Figure 9.**
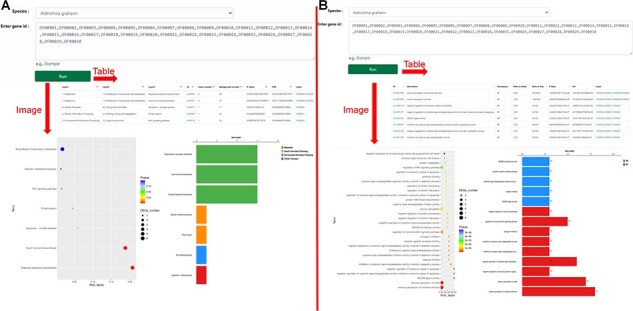
‘KEGG Enrichment’ and ‘Go Enrichment’. (**A**) Gene function annotation analysis and enrichment analysis on KEGG. (**B**) Gene function annotation analysis and enrichment analysis on GO.

**Figure 10.**
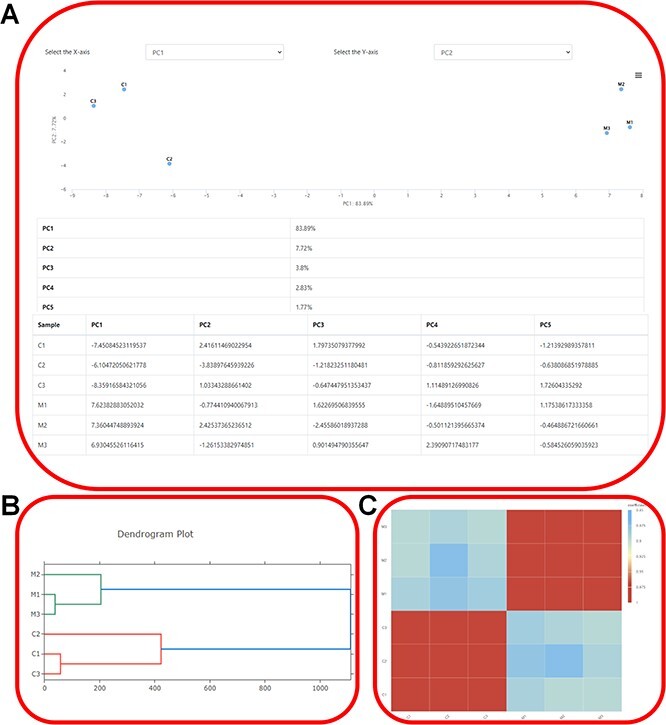
Expression profiling. (**A**) Showcasing the distribution of samples in the principal components and elucidating the contributions of each principal component using ‘PCA Analysis’. (**B**) The dendrogram depicting the hierarchical clustering results of transcriptome samples. (**C**) Visualization of gene expression correlations using ‘Correlation Heatmap’.

The tool module contains several utility tools, including ‘Muscle’, ‘Genewise’, ‘Primer’, ‘Lastz’, ‘Go Enrichment’ and ‘KEGG Enrichment’, which allow relatively complete bioinformatics analysis (Figures 8 and 9). Utilities, including ‘PCA Analysis’, ‘Hcluster’ and ‘Correlation Heatmap’, allow uploading expression information files for mapping and analysis (Figure 10). ‘Muscle’ is a multi-sequence alignment tool that can obtain homology between genes and construct intuitive phylogenetic tree maps. By using the ‘Lastz’ and ‘GeneWise’ tools, users can complete genome alignment and gene homology annotation, respectively. Users can click ‘Primer’, input the nucleic acid sequence or select the sequence range, adjust the corresponding parameters and complete the specific primer design for functional gene cloning. Users can select the KEGG database or GO database for functional cluster analysis, input gene ID in the dialog box and screen the functional genes of interest according to the transcriptome data of necrophagous insects (Figure 9).

### Case base

**Figure 11.**
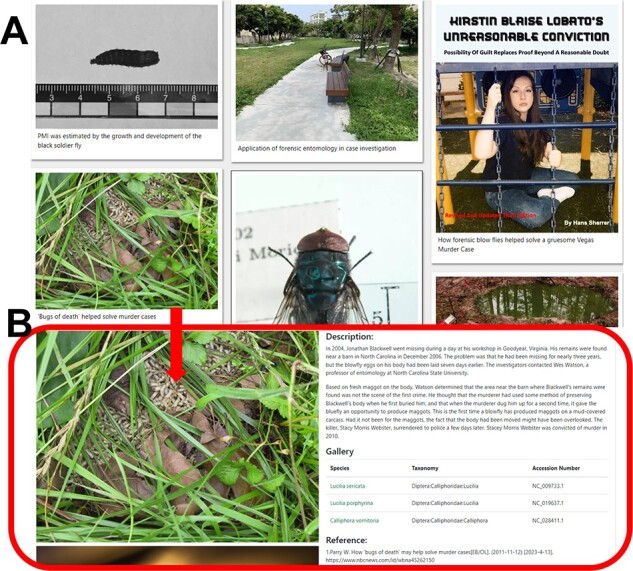
Detailed description of the ‘Case base’ module. (**A**) Case picture introduction. (**B**) Details of the case and the necrophagous insects involved.

The Case base module shows a variety of cases related to forensic entomology. The user can click on the cover image to enter the details page, which displays case details, pictures and references (Figure 11).

## Discussion

With the significant role of necrophagous insects in forensic investigation and the rapid advancements in sequencing technology, a forensic entomological database was urgently required. Thus, HOFE emerged as the first comprehensive and interactive forensic entomological database. HOFE consolidates multi-omics and biological data related to forensically important species, presenting these resources through an integrated and user-friendly platform. For the first time, this platform provides a repository of critical species-related data, including genetic information, morphological imagery and real-world case studies. This database can be used to further explore the developmental mechanism and biological characteristics of necrophagous insects and provide more accurate data support for the study of entomological evidence (45, 46). Users can selectively choose species of interest and obtain relevant information such as genomic sequences and genomic maps through online browsing and searching. Additionally, users can analyze or upload their own data online via the website. Through the operation of the intelligent and interactive platform interface, criminal technicians can integrate morphology and omics indicators of necrophagous insects in case practice to provide evidence for court investigations. By collecting numerous cases, HOFE can provide forensic experts with information on the processes, methods and techniques of insect species identification and improve their ability to analyze cases using necrophagous insects.

Among the datasets, the genetic resource of Diptera was the largest, and the data of Calliphoridae and Muscidae were relatively rich. However, the genomic data of Coleoptera need to be supplemented. We will update our data resource in a timely manner when new omics data of necrophagous insects are available or published, by using public bioinformatics databases and our submission feature. In addition, we will continue to collect cases with multiple insects from journals, news reports and our’ practice to enrich case base. Meanwhile, our research team are undertaking more forensically important sequencing projects to contribute more genomic data for forensic entomology study.

## Data Availability

All data in HOFE are stored in the download section, and users can download it on demand.
